# Cost-effectiveness analysis of Vaborem in Carbapenem-resistant Enterobacterales (CRE) -*Klebsiella pneumoniae* infections in Italy

**DOI:** 10.1186/s13561-021-00341-z

**Published:** 2021-10-30

**Authors:** Francesco Saverio Mennini, Mario Gori, Ioanna Vlachaki, Francesca Fiorentino, Paola La Malfa, Duccio Urbinati, Massimo Andreoni

**Affiliations:** 1 EEHTA CEIS, Faculty of Economics, University of Rome “Tor Vergata”, Rome, Italy; 2 Institute of Leadership and Management in Health, Kingston University, London, UK; 3 Menarini Ricerche SpA, Florence, Italy; 4 IQVIA, Milan, Italy; 5 Faculty of Medicine, University of Rome “Tor Vergata”, Rome, Italy; 6 Infectious Disease Unit, Policlinic Hospital of Rome “Tor Vergata”, Rome, Italy

## Abstract

**Background:**

Vaborem is a fixed dose combination of vaborbactam and meropenem with potent activity against target Carbapenem-resistant *Enterobacterales* (CRE) pathogens, optimally developed for *Klebsiella pneumoniae* carbapenemase (KPC). The study aims to evaluate the cost-effectiveness of Vaborem versus best available therapy (BAT) for the treatment of patients with CRE-KPC associated infections in the Italian setting.

**Methods:**

A cost-effectiveness analysis was conducted based on a decision tree model that simulates the clinical pathway followed by physicians treating patients with a confirmed CRE-KPC infection in a 5-year time horizon. The Italian National Health System perspective was adopted with a 3% discount rate. The clinical inputs were mostly sourced from the phase 3, randomised, clinical trial (TANGO II). Unit costs were retrieved from the Italian official drug pricing list and legislation, while patient resource use was validated by a national expert. Model outcomes included life years (LYs) and quality adjusted life years (QALYs) gained, incremental costs, incremental cost-effectiveness ratio (ICER) and incremental cost-utility ratio (ICUR). Deterministic and probabilistic sensitivity analyses were also performed.

**Results:**

Vaborem is expected to decrease the burden associated with treatment failure and reduce the need for chronic renal replacement therapy while costs related to drug acquisition and long-term care (due to higher survival) may increase. Treatment with Vaborem versus BAT leads to a gain of 0.475 LYs, 0.384 QALYs, and incremental costs of €3549, resulting in an ICER and ICUR of €7473/LY and €9246/QALY, respectively. Sensitivity analyses proved the robustness of the model and also revealed that the probability of Vaborem being cost-effective reaches 90% when willingness to pay is €15,850/QALY.

**Conclusions:**

In the Italian setting, the introduction of Vaborem will lead to a substantial increase in the quality of life together with a minimal cost impact, therefore Vaborem is expected to be a cost-effective strategy compared to BAT.

**Supplementary Information:**

The online version contains supplementary material available at 10.1186/s13561-021-00341-z.

## Background

Carbapenamases are versatile β-lactamase with an ability to hydrolyse a wide spectrum of β-lactams such as penicillins, cephalosporins, monobactams, and carbapenems [1–4]. Hence, the bacteria producing these enzymes are resistant to a broad spectrum of antibiotics, posing a considerable challenge in their treatment. Carbapenamases are mostly found in gram negative bacteria belonging to the taxonomically diverse family of Enterobacterales (previously classified as Enterobacteriacea) and are called Carbapenem-resistant Enterobacterales (CRE) [5]. A recent study in Europe reported that CRE infections have been expanding rapidly and led to the development of serious infections resulting in significant morbidity and mortality [6]. *Klebsiella pneumoniae* Carbapenemase (KPC) producers are a high priority and high risk CRE enlisted by the World Health Organization and represent the fastest growing antibiotic resistance threat in Europe [6–8]. The population-weighted mean carbapenem resistance percentage during the period of 2015–2018 in Europe is between 6.8–7.5%, being much higher in Italy (33.5–26.8%) [9, 10]. The fatality associated with *K. pneumoniae* resistant to carbapenems has risen up to six-fold between 2007 and 2015 [9, 10].

The infections caused by *K. pneumoniae* include urinary tract infections, lower respiratory tract infections, intra-abdominal infections, and bloodstream infections among others. The CRE-KPC infections are difficult to treat as carbapenem resistance is most often accompanied by multi-drug resistance [8, 11]. With the increasing rate of infections and lack of effective treatment options emphasis is on the discovery of new antibiotics to address the present challenge. Traditionally, the antibiotics belonging to either polymyxin (e.g.*,* colistin or polymyxin B) or aminoglycoside (amikacin, tobramycin, gentamicin) classes have been prescribed for CRE infections. However, these antibiotics have some safety concerns associated with toxicity and more importantly the CRE-KPC has developed resistance toward these antibiotics reducing the efficacy of treatment [12]. Even combination therapies with traditional antibiotics have been unable to keep pace with the rapid evolution of the CRE-KPC infections [12]. Newer antibiotics and combinations which include for example tigecycline and ceftazidime-avibactam are presently being used as alternative treatment options. These alternate treatment options have fewer side effects compared to traditional drugs, but further real-world evidence needs to be established to validate the effectiveness of these drugs [12].

Meropenem–vaborbactam, with the brand name Vaborem, is the first drug combination that includes a boronic acid-based beta-lactamase inhibitor and a carbapenem [13, 14]. Vaborbactam is a potent inhibitor of class A serine carbapenemase specifically developed to inhibit KPC enzyme. Given its β-lactamase inhibition profile, vaborbactam extends the spectrum of activity of meropenem to strains of *Enterobacterales* producing KPC-type and other class A serine carbapenemases. Meropenem is a broad-spectrum carbapenem antibiotic that has been used worldwide for over two decades for the treatment of serious infections [13, 14].

Vaborem has shown positive results in two phase 3 clinical trials. In particular in the TANGO II trial, a randomised, prospective, pathogen-specific multicentre phase 3 clinical trial versus Best Available Treatment (BAT). Vaborem has shown higher clinical cure rates, lower nephrotoxicity and reduction in mortality rates compared to treatments currently used in clinical practice [15]. The importance of Vaborem in the treatment of CRE infections has been acknowledged by the World Health Organization (WHO) by its inclusion in the 21st WHO model list of essential medicines [16].

However, the economic impact of introducing Vaborem on healthcare expenditure has not yet been investigated. From an Italian perspective, the guidelines published by the Italian Medicines Agency highly recommend performing economic evaluation analyses on any new drugs/active ingredients or drugs with extended indication [17]. Cost-effectiveness analysis has been frequently used as an efficient tool in the economic evaluation of new antibiotics in establishing value-based pricing [18]. In the present study we intend to assess the cost-effectiveness of Vaborem versus BAT for the treatment of patients with CRE-KPC associated infections from the perspective of National Health Service (NHS), in Italy.

## Methods

### Model structure

**Fig. 1:**
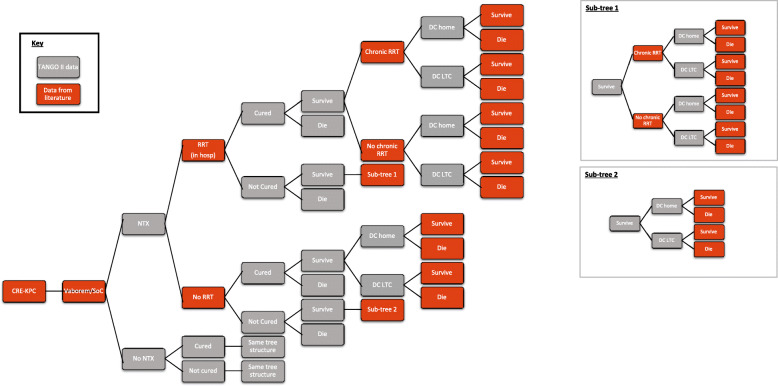
Decision tree model structure. BAT: best available therapy; CRE: Carbapenem-Resistant *Enterobacterales*; DC: discharged; LTC: long-term care; NTX: nephrotoxicity; RRT: renal replacement therapy. Probabilities of transition between health states are reported in Table 1

A cost-effectiveness analysis (CEA) was conducted based on a decision tree model that simulates a scenario with Vaborem and one with BAT (without Vaborem) in the treatment of CRE-KPC infections. Decision tree model was chosen as they are particularly suited to modelling acute care decision problems and medium-term diseases, such as infections. The model structure, presented in Fig. 1, simulates the clinical pathway followed by patients with a confirmed CRE-KPC infection.

Two cohorts were considered in the model, one for each scenario, with 1000 hospitalised patients, representative of the target population, with confirmed diagnosis of CRE-KPC infection. The model captured short-term outcomes until day 28 (in line with the TANGO II study protocol) [15] and simulated long-term outcomes (5 years). In line with the Italian guidelines [17], the benefits and costs were discounted at a rate of 3%. The total costs and quality adjusted life years (QALYs) were calculated based on the occurrence of events. These were then simulated over the model time horizon to calculate total costs and QALYs for the two cohorts from which incremental results and the cost per QALY were determined.

#### Patient population

The target population included adult patients ≥ 18 years with CRE-KPC associated infections. The CRE-KPC infections considered were the same of TANGO II [15]: complicated urinary tract infections (cUTI), including acute pyelonephritis (AP), hospital acquired/ventilator-associated bacterial pneumonia (HABP/VABP), bacteraemia, or complicated intra-abdominal infection (cIAI), that occurs in association with, or is suspected to be associated with, any of these infections. The baseline characteristics of the patients included in the CEA model was aligned to the patient demographics from the microbiologic-CRE-modified intent-to-treat (mCRE-MITT) population (*n* = 47) of TANGO II in which most patients had CRE-KPC associated infections (87.2%, *n* = 41). In particular, patients’ mean age and weight were 62.5 years and 76 kg and 51.1% of patients were male [15].

#### Time horizon

The time horizon of the CEA was 5 years, which was considered sufficient to capture the main differences among the two scenarios in terms of costs and outcomes. This time horizon was also in line with the Italian guidelines’ requirements [17] and other published economic studies on CRE infections that considered a time horizon of 5 years or shorter [23, 24]. Different time horizons were selected in the sensitivity analysis.

#### Study perspective

In the analysis, the Italian NHS perspective was adopted. This option was considered conservative because only direct costs borne by the NHS for the management of CRE-KPC associated infections were included. The indirect costs such as loss of productivity of patients and their caregivers were not considered although their burden is expected to decrease with the reduction of disease complications. Further, the expected reduction in the utilisation of resources during hospitalisation (e.g. with the reduction of septic shock) was not valued because in-hospital episodes are financed through all-inclusive tariffs (e.g. diagnostic-related groups, DRG). The direct costs considered in this study included treatment costs, administration costs, disease management costs, disease complication costs and treatment-related adverse event costs.

### Model inputs and data sources

#### Clinical inputs

##### Treatment efficacy

The CEA compared Vaborem with BAT as the main standard of care comparator in line with the TANGO II study and confirmed by an Italian medical expert. BAT includes (alone or in combination): carbapenem, aminoglycoside, polymyxin B, colistin, tigecycline or ceftazidime-avibactam (monotherapy only) [15]. A majority of patients 67% in the mCRE-MITT population administered a BAT regimen received combination therapy, usually including a carbapenem agent. Supplementary Table 1 provides a breakdown of BAT as per the TANGO II study. The clinical effectiveness of treatment with Vaborem was factored in terms of cure rate, mortality, treatment-emergent adverse effects, and nephrotoxicity probability. These data were mainly retrieved from the TANGO II study [15, 19]. The probability of renal complications among patients with nephrotoxicity were obtained from the medical expert. The factors considered to estimate the mortality after 28 days in patients without and with chronic renal replacement therapy (RRT) included: demographics of eligible population (age and sex), Charlson Comorbidity Index (CCI) of patients in TANGO II study, relative hazard ratio associated to CCI retrieved from literature [21], general population mortality rate in Italy [20] and mortality rate associated with patients who underwent RRT [22] (Table 1).

**Table 1:** Summary of inputs and data sources used

Treatment	Vaborem	BAT	Comments and sources
**Efficacy**			
Cure	59.4%.	26.7%	TANGO II [15]
Mortality at day 28	15.6%	33.3%	TANGO II [15]
**Probability of discharge**			
At home	77.3%	77.3%	TANGO II, Menarini data on file [15, 19]
For long-term care	22.7%	22.7%	TANGO II, Menarini data on file [15, 19]
**Probability of complication**			
Septic shock	3.1%	26.7%	TANGO II, Menarini data on file [15, 19]
Nephrotoxicity	3.1%	26.7%	TANGO II, Menarini data on file [15, 19]
RRT (inpatient)	25.0%	25.0%	Italian medical expert opinion. Applied to patients with nephrotoxicity
RRT (after discharge)	40.0%	40.0%	Italian medical expert opinion. Applied to patients who have received RRT even during admission.
**Mortality for all causes (after 28 days)– Without Chronic RRT**			
Year 1	29.6%	29.6%	Calculated considering: • Patients’ characteristics from TANGO II (sex and age) [15] • Italian mortality tables [20] • Proportion of patients with low (1–2), medium (3–4) and high (≥5) CCI of 10.6, 10.6 and 78.7%, respectively (TANGO II) [15] • HR as per CCI level [21]
Year 2	30.3%	30.3%	
Year 3	31.0%	31.0%	
Year 4	31.8%	31.8%	
Year 5	32.6%	32.6%	
**Mortality for all causes (after 28 days)– With Chronic RRT**			
Year 1	61.4%	61.4%	Calculated considering: • Patients’ characteristics from TANGO II (sex and age) [15] • Italian mortality tables [20] • Proportion of patients with low (1–2), medium (3–4) and high (≥5) CCI of 10.6, 10.6 and 78.7%, respectively (TANGO II) [15] • Scottish register of RRT patients [22]
Year 2	70.3%	70.3%	
Year 3	75.4%	75.4%	
Year 4	89.2%	89.2%	
Year 5	92.4%	92.4%	

BAT: Best Available Therapy; mCRE-MITT: microbiologic-CRE-modified intent-to-treat; RRT: Renal Replacement Therapy; HR: Hazard Ratio; CCI: Charlson Comorbidity Index

##### Quality of life (QoL)

Since QoL data were not collected during the TANGO II study [15], health utilities were retrieved from literature [23–27]. The health utilities considered in the analysis were associated to the following health states: (i) hospitalisation without nephrotoxicity [27], (ii) hospitalisation with nephrotoxicity [25], (iii) acute RRT [25], (iv) chronic RRT [26], (v) discharged home [23] and (vi) discharged to long-term care (LTC) [28]. The same utility values were considered in a previously published economic evaluation of CRE population [24]. The duration of hospitalisation was estimated considering the reported DRG codes of interest [29], while the duration of nephrotoxicity (28 days) and acute RRT (90 days) was estimated based on a prospective study [30]. The duration of chronic RRT, discharge at home and LTC depends on the patient’s path and was therefore calculated in a residual manner (Supplementary Table 2).

#### Cost inputs

The costs considered in the CEA were associated with: (i) pharmacological treatment (drug acquisition), (ii) management of the infections during hospitalisation, and (iii) complications associated with the infections such as: therapeutic failure, chronic RRT, and LTC. The costs associated with drug administration, nephrotoxicity, and the use of RRT during the first hospitalisation were not considered in the analysis as they were already included in the all-inclusive DRG tariff for hospitalisation.

##### Treatment cost

At the beginning of the model, all patients received one course of either Vaborem, or BAT based on their treatment group. The acquisition costs for the BAT were estimated based on the ex-factory price without value-added tax after applying lawful discounts [31]. All other data such as the dosage, average duration of treatment and patient distribution among the different treatment options required for calculating the treatment costs were retrieved from the TANGO II study [15]. The cost for a complete cycle of treatment with Vaborem and BAT was estimated as € 2301.49 and €1485.23, respectively (Supplementary Table 3 and Supplementary Table 6).

##### Disease management cost

The disease management costs are associated with the hospitalisation stay. The in-hospital costs were estimated at € 4533.27 based on the National DRG tariffs [32], which are comprehensive and include all costs incurred during hospitalisation. Therefore, in order to avoid double counting, the cost of drug acquisition with BAT (€ 1485.23) was subtracted from the cost of hospitalisation estimated based on DRG’s all-inclusive tariff (€ 4533.27). The average duration and cost of hospitalisation was estimated to be 12.2 days and € 3048.04 respectively at baseline. Different tariffs were selected in the sensitivity analysis (Supplementary Table 4).

##### Disease complication cost

The model assumes that upon treatment failure patients received a second course of antibiotic. The estimated cost for the second course of therapy, independently of the treatment arm, was considered to be equal to BAT in terms of both drug acquisition costs and hospitalisation costs (€1,485.23 and €3048.04, respectively). The annual cost for chronic RRT (that included hospitalisation, dialysis, diagnostic procedure and drugs) was extracted from a retrospective study in an Italian cohort and was estimated to be €38,819.40 [33]. The cost associated with LTC was calculated based on the unit cost from national tariffs [34] and applied to the proportion of patients requiring it estimated from TANGO II study [15]. The annual cost of LTC was estimated to be €45,213.7 in the first year and €44,268.3 from the second year onwards [34] (Supplementary Table 5 and Supplementary Table 6).

### Sensitivity analysis

Deterministic and probabilistic sensitivity analyses were performed to explore the level of uncertainty in the model results. In order to assess the robustness of the baseline scenario results, deterministic sensitivity analyses were carried out in which the most uncertain parameters were varied. The uncertain parameters included the demographics characteristics of Italian multicentre study conducted on patients with CRE-KPC infections (mean age–68 years and 63% of patients were male) [35], shorter time horizon (28-days and one year), different discount rates (0 and 5%) [17] and a follow-up cost after home discharge (a medical visit and a complete blood count). The sensitivity analyses were also performed with a ± 20% mortality rate at 28 days, ±20% RRT costs, ±20% utility values, LTC for 50% patients and with alternative hospitalisation cost (€ 4981.23).

In the probabilistic sensitivity analysis 1000 random extractions (Monte Carlo simulations) of the model’s inputs were simulated. The gamma distribution was considered for the continuous and positive variables (i.e. age, weight, cost, and duration of hospitalisation) and the beta distribution for the variables that assumed values between 0 and 1 (i.e. probability, utility, proportion of men). For the efficacy variables coming from TANGO II, the relative standard errors were considered and for the other parameters a standard error of 20% of the average value was considered. Mean incremental results were recorded and illustrated through an incremental cost-effectiveness plane and a cost-effectiveness acceptability curve (CEAC) was also plotted.

## Results

### Base case analysis

Over a 5-year time horizon, the cohort receiving Vaborem accrued 1.786 QALYs at a cost of €29,750.85, while patients receiving BAT accrued 1.403 QALYs at a cost of €26,202.28. Hence, the increase in costs associated with Vaborem versus BAT treatment for a representative patient with CRE-KPC infection was €3548.57. This increase in cost is mainly attributable to the costs of the treatment drug and LTC associated with higher survivability (Table 2). In terms of efficacy, the model estimated an increase in QALY of 0.384 (quality adjusted years of life gained) and LY of 0.475 (years of life gained). In a specular way, the majority of QALY increase is associated to the longer survival (QALYs increase by 30% after hospital discharge), while QALYs associated to RRT and nephrotoxicity significantly decrease due to the reduction of probability of disease complications. Detailed results in terms of costs and effectiveness are reported in Table 2. Based on treatment cost and effectiveness, an incremental cost-utility ratio (ICUR) of € 9246/QALY and an incremental cost-effectiveness ratio (ICER) of € 7473/LY were obtained.

**Table 2:** Results of base case analysis

	Vaborem	BAT	Difference	Percentage change
**Costs (€)**				
**Total Costs**	**29,750.85**	**26,202.28**	**3548.57**	**13.5%**
Pharmacological treatment	2301.49	1485.23	816.26	55.0%
Admission	3048.04	3048.04	0.00	0.0%
Therapeutic failure	1841.64	3324.40	− 1482.76	−44.6%
Chronic RRT	129.49	873.10	− 743.61	− 85.2%
Long-term care	22,430.18	17,471.51	4958.68	28.4%
**Clinical Effectiveness**				
**Total QALY**	**1.786**	**1.403**	**0.384**	**27.4%**
Nephrotoxicity-free hospitalisation	0.033	0.031	0.002	7.2%
Hospitalisation with nephrotoxicity	0.002	0.013	−0.012	− 88.3%
Acute RRT	0.000	0.002	−0.002	−85.2%
Chronic RRT (after 90 days)	0.002	0.013	−0.011	−85.2%
After Hospital discharge	1.749	1.343	0.406	30.3%
**Total LYs**	**2.253**	**1.778**	**0.475**	**26.7%**

BAT: best available therapy; LY: life years; QALY: quality assisted life years; RRT: renal replacement therapy

### Deterministic sensitivity analyses

The deterministic sensitivity analyses performed on most uncertain parameters is presented in Table 3 and illustrated in Supplementary Fig. 1. The results were most sensitive to LTC (when set to 50%) where the estimated ICUR was €26,691/QALY. A relevant impact was also observed when shorter time horizons were considered, with Vaborem being dominant when 28 days time horizon and with an estimated ICUR of €5316/QALY when 1 year time horizon was considered. The improvement of results when assessing shorter time horizons is associated to the partial or complete exclusion of long-term care costs. Symmetrically, when survival costs were increased because of the inclusion of follow-up costs after home discharge, ICUR slightly worsened (€9276/QALY). In the vial sharing scenario, where BAT acquisition costs were reduced because of the zero waste assumption, results worsened (€ 9859/QALY) while higher hospitalisation cost improved them (€7500/QALY). Due to the expected higher survival of patients treated with Vaborem, the increase of utility levels had a positive impacted on the results (€7758/QALY) while their reduction had a negative impact (€11,558/QALY). Finally, calibrating with different patients’ characteristics based on published literature [35] did not significantly impact results (€9269/QALY). Overall, the sensitivity analyses revealed that the base case scenario is robust, and all tested scenarios always remained below the threshold value of €30,000/QALY.

**Table 3:** Results of deterministic sensitivity analysis

Input	*Δ* Costs (€)	*Δ* LY	*Δ* QALY	ICER (€/LY)	ICUR (€/QALY)
**Base case scenario**	3549	0.475	0.384	7473	9246
**Model settings**					
Discount rate 0%	3733	0.494	0.399	7551	9347
Discount rate 5%	3436	0.463	0.374	7423	9181
Time horizon 28 days	− 666,50	0.000	0.002	Dominant	Dominant
Time horizon 1 year	717	0.164	0.135	4384	5316
Alternative patients’ demographic characteristics*	3247	0.433	0.350	7491	9269
Utility Values + 20%	3549	0.475	0.457	7473	7758
Utility Values −20%	3549	0.475	0.307	7473	11,558
**Calibration of effectiveness**					
Long-term care (50%)	9499	0.475	0.356	20,004	26,691
Mortality after 28 days + 20%	3025	0.423	0.342	7154	8833
Mortality after 28 days −20%	4150	0.535	0.431	7762	9622
**Calibration of costs**					
Hospitalisation costs € 4981.23	2878	0.475	0.384	6061	7500
RRT costs + 20%	3697	0.475	0.384	7786	9634
RRT costs −20%	3400	0.475	0.384	7160	8859
Vials sharing	3784	0.475	0.384	7968	9859
Follow-up costs after home discharge € 87.41	3560	0.475	0.384	7497	9276

*Patients with an average age of 68.0 years and predominantly male (63.1%) [34]

BAT: best available therapy; ICER: incremental cost-effectiveness ratio; ICUR: incremental cost-utility ratio; LY: life years; QALY: quality assisted life years; RRT: renal replacement therapy

### Probabilistic sensitivity analyses

**Fig. 2:**
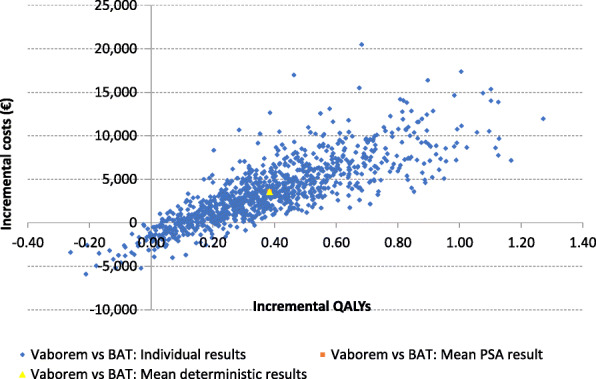
Incremental cost-effectiveness plane. BAT: best available therapy; QALY: quality adjusted life year; PSA: probability sensitivity analysis

An estimated average cost increase with Vaborem compared to BAT was €3579 and a gain in terms of QALY was 0.383 with a probabilistic ICUR of € 9342/QALY. The incremental cost-effectiveness plane showed that 85.9% of the iterations were in the north-east quadrant where Vaborem is more costly and more effective than BAT and 11.2% fell in the south-east quadrant where Vaborem is less costly and more effective. No simulation was observed in the north-west quadrant where the change in costs were positive and the benefits were negative (Fig. 2).

**Fig. 3:**
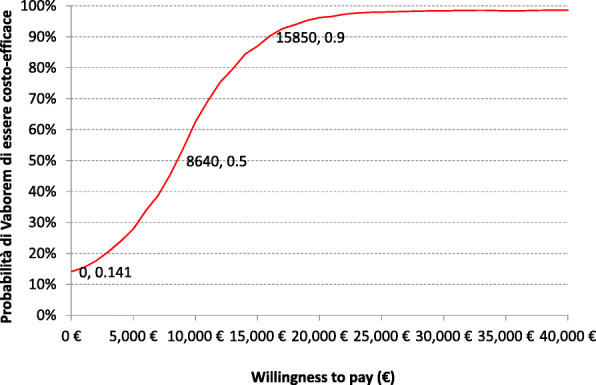
Cost-effectiveness acceptability curve

The data from the probabilistic simulations were further used to generate a CEAC at different levels of willingness to pay. The CEAC illustrates the probability of Vaborem being cost-effective compared to BAT, at various willingness to pay thresholds. At the willingness to pay thresholds of €8640/QALY, the probability of Vaborem being cost-effective compared to BAT reaches 50% and at a threshold of €15,850/QALY, the cost-effectiveness of Vaborem reaches 90% (Fig. 3).

## Discussion

From an Italian perspective, the treatment of CRE-KPC associated infection with Vaborem may be a cost-effective option considering a threshold of € 9246/QALY gained, which is much below the commonly accepted threshold value of € 30,000/QALY – € 50,000/QALY. It is however important to note that an official cost-effectiveness threshold has not yet been defined in Italy [36, 37]. Overall, in the current study, the cost-effectiveness model estimated that, over a 5-year time horizon, the increase in discounted costs associated with Vaborem versus BAT treatment for a representative patient with CRE-KPC infection is € 3548.57. This increase is mainly attributable to the costs of drug treatment and LTC associated with higher survivability. In particular, the higher cost of the LTC is due to the higher survival of patients treated with Vaborem. On the other hand, the costs associated with therapeutic failure and RRT are expected to decrease as a result of the increased efficacy and improved safety profile of Vaborem in comparison to BAT. From the point of view of effectiveness, the model estimated an increase in QALYs gained of 0.384 and an increase in LYs gained of 0.475. The increase in QALYs is mainly associated with increased survival after hospital discharge and reduced hospital stay with nephrotoxicity and reduced need for RRT. In the current scenario, with limited alternatives for the treatment of CRE-KPC associated infection, a potentially cost-effective treatment with Vaborem will be an important addition to curtail the growing incidence of this infection [9, 10].

The model used in the present study is based on a decision tree structure that simulated the clinical pathway followed by patients with a confirmed CRE-KPC infection in line with the TANGO II study. This simple model is the most appropriate in the area of infectious diseases, when interaction between individuals is not considered relevant [23]. This model approach has also been adopted in other two cost-utility studies conducted in individuals with CRE associated infections [23, 24]. Further, the population of TANGO II, considered in the model, is aligned with the population reported in real-world evidence studies in terms of type of infection, age and sex distribution [35, 38].

To the best of our knowledge, the current study is the first to assess the cost-effectiveness of Vaborem for the treatment of adult patients with CRE-KPC associated infections. The key strength of the current study is the enriched patient population which included the majority of CRE infections such as cUTI, cIAI, HABP, VABP and bacteraemia.

The analysis may be deemed as conservative for several reasons. First, only direct costs borne by the National Health System were considered neglecting the indirect costs, which include loss of productivity of patients and their caregivers and which are expected to decrease with the reduction of disease complications. Second, the savings associated with lower intensity of in-hospital care utilisation are not valued in the analysis, for instance the savings associated to the reduction of treatment-emergent adverse events (diarrhoea, anaemia, hypotension, sepsis, septic shock and acute renal failure) were not valued in the analysis since costs were estimated based on omni-comprehensive DRG tariffs. Indeed, in TANGO II the only treatment-emergent adverse events with higher incidence for patients treated with Vaborem than BAT was hypokalaemia [15]. Third, the DRG tariffs used to estimate the costs associated to the disease management may substantially underestimate the actual costs borne by the NHS as emerged in other Italian studies [39–41]. Finally, the model does not account for other advantages associated to the introduction of a new antibiotic such as preventing the transmission of infections to other patients and slowing down the development of resistance to other drugs.

The study also presents some limitations. A major limitation of the study is that model inputs were mainly estimated based on the TANGO II study, in which the sample size of microbiologic-CRE-modified intent-to-treat (mCRE-MITT) was small (*N* = 47) [15]. Still, TANGO II is a phase 3, randomized, prospective, multicenter, multinational, open-label, active-controlled clinical trial [15] performed in very severe life threatening infections where patients enrolment may be considered particularly challenging given that enrolment period lasted over two years. Despite the uncertainty of the model clinical inputs, probabilistic sensitivity analysis consistently leads to positive results with the probability of Vaborem being cost-effective over 90% considering a willingness to pay of €16,000/QALY.

Additionally, in the TANGO II study the follow-up period was relatively short (28 days) leading to the need to integrate its data from information retrieved from published literature and medical experts’ opinion in order to simulate data for a longer time horizon (5-years). The uncertainty associated to longer time horizon of the model (5-year) compared to the clinical trial follow-up (28 days) was assessed in a scenario analysis resulting in better results.

Furthermore, during the sensitivity analysis the possibility of carrying out an indirect comparison analysis to compare the effectiveness of Vaborem [15] versus ceftazidime-avibactam [42] was evaluated, but it was not considered possible due to the high heterogeneity in the design of the studies in terms of patient population (e.g. eligible population, infection type, previous antibiotic use and region), the definition of BAT treatment regimen and the outcomes considered.

## Conclusion

The inclusion of Vaborem into the treatment armamentarium for serious CRE-KPC infections is expected to lead to a significant improvement in the clinical cure rates, while lowering nephrotoxicity rates and mortality. From this study it emerges that, in the Italian setting, the introduction of Vaborem will lead to a substantial increase in the quality of life together with a minimal cost impact. Hence, Vaborem is expected to be a cost-effective treatment strategy compared to BAT for the treatment of CRE-KPC infections.

## Supplementary Information

**Additional file 1.**

## Data Availability

All data generated or analysed during this study is included in this published article (and its supplementary information files).

## Abbreviations

AP: Acute pyelonephritis
BAT: Best available therapy
CCI: Charlson Comorbidity Index
CEA: Cost-effectiveness analysis
CEAC: Cost-effectiveness acceptability curve
CRE: Carbapenem-resistant Enterobacterales
DRG: Diagnostic-related groups
ECDC: European Centre for Disease Prevention and Control
HTA: Health technology assessment
ICER: Incremental cost-effectiveness ratio
ICUR: Incremental cost-utility ratio
KPC: *Klebsiella pneumoniae* carbapenemase
LTC: Long-term care
LY: Life years
NHS: National Health Service
QALY: Quality adjusted life years
QoL: Quality of life
RRT: Renal replacement therapy
WHO: World Health Organization
