# Supplementary material for: Cost-effectiveness analysis of Vaborem in Carbapenem-resistant Enterobacterales (CRE) -Klebsiella pneumoniae infections in Italy
Source: Health Econ Rev. 2021 Oct 30;11:42. doi: 10.1186/s13561-021-00341-z (PMC8557067; doi:10.1186/s13561-021-00341-z)
Supplement: Supplementary file 1 — Additional file 1. [file 13561_2021_341_MOESM1_ESM.docx]

SUPPLEMENTARY MATERIAL

**Supplementary Table 1: Comparative treatment (best available therapy) identified for comparative economic analysis***

| **Treatment** | **Patients (%)** |
| --- | --- |
| **Monotherapy** | **26.7** |
| Aminoglycosides | 6.7 |
| Carbapenems | 6.7 |
| Ceftazidime-Avibactam | 6.7 |
| Polymyxin/Colistin | 6.7 |
| **Combination of two therapies** | **46.7** |
| Carbapenems + Aminoglycosides | 6.7 |
| Carbapenems + Polymyxin/Colistin | 6.7 |
| Carbapenems + Tigecycline | 13.3 |
| Polymyxin/Colistin + Aminoglycosides | 20.0 |
| **Combination of three therapies** | **13.3** |
| Carbapenems + Polymyxin/Colistin + Tigecycline | 6.7 |
| Carbapenems + Polymyxin/Colistin + Ceftazidime-Avibactam | 6.7 |
| **Combination of four therapies** | **13.3** |
| Carbapenems + Polymyxin/Colistin + Aminoglycosides + Tigecycline | 13.3 |
| **Total** | **100.00** |

*Data in the table has been extracted from the TANGO II study [1]

**Supplementary Table 2: Quality of life data**

| **Utility** | **Value** | **Source** | **Duration (days)** | | **Source** | |
| --- | --- | --- | --- | --- | --- | --- |
| **Short period** | | | | | |  |
| Nephrotoxicity-free hospitalisation | 0.73 | Tsevat 1995 [2] | 12.20 | Activity SDO 2018 [3] | |  |
| Hospitalisation with nephrotoxicity | 0.66 | Faulhaber-Walter 2016 [4] | 28.00 | Prescott 2007 [5] | |  |
| Acute RRT | 0.66 | Faulhaber-Walter 2016 [4] | 62.00 | Prescott 2007 [5] | |  |
| **Long period** |  |  |  |  | |  |
| Chronic RRT (after 90 days) | 0.59 | Liem 2008 [6] | NA | Patient path dependent variable | |  |
| Discharge at home | 0.84 | Bartsch 2017 [7] | NA | Patient path dependent variable | |  |
| Long-term assistance | 0.64 | MacNeil Vroomen 2012 [8] | NA | Patient path dependent variable | |  |

RRT: Renal replacement therapy

**Supplementary Table 3: Acquisition costs associated with treatment with Vaborem and BAT**

| **Class** | **Drug** | **mg per *vial*** | **Dose (mg)** | **Vials per somm.ne** | **Daily frequency** | **Days of treatment** | **Cost per vial^ [9](€)** | **Cost per treatment**  **(€)** |  |
| --- | --- | --- | --- | --- | --- | --- | --- | --- | --- |
| **Vaborem** | | | | | | | | |  |
| Carbapenem + inhibitory. β-lactamase | Vaborem | 1,000 | 2,000 | 1.5 | 3 | 8.5 | 60.17 | 2,301.49 |  |
| **Best available therapy** | | | | | | | | | |
| Aminoglycosides | Gentamicin | 80 | 382 | 5 | 1 | 6 | 0.54 | 16.20 |  |
| Carbapenems | Ertapenem | 1,000 | 1,000 | 1 | 1 | 1 | 40.61 | 798.77 |  |
|  | Meropenem | 500 | 1,278 | 3 | 3 | 13 | 6.48 |  |  |
| Ceftazidime-Avibactam | Ceftazim | 2,000 | 2,000 | 1 | 3 | 8 | 100.02 | 2,400.48 |  |
|  | Avibactam | 500 | 500 | 1 |  | 8 |  |  |  |
| Colistin | Colistin | 2 MU | 4.4 MU | 3 | 2 | 3 | 24.98 | 449.64 |  |
| Carbapenems + aminoglycosides | Gentamicin | 80 | 382 | 5 | 1 | 7 | 0.54 | 427.14 |  |
|  | Meropenem | 500 | 1,278 | 3 | 3 | 7 | 6.48 |  |  |
| Carbapenems + colistin | Ertapenem | 1,000 | 1,000 | 1 | 1 | 4 | 40.61 | 1,053.56 |  |
|  | Meropenem | 500 | 1,278 | 3 | 3 | 5 | 6.48 |  |  |
|  | Colistin | 2 MU | 4.4 MU | 3 | 2 | 4 | 24.98 |  |  |
| Carbapenems + tigecycline | Meropenem | 500 | 1,278 | 3 | 3 | 5.5 | 6.48 | 1,045.00 |  |
|  | Tigecycline | 50 | 78 | 2 | 2 | 5.5 | 32.92 |  |  |
| Colistin + aminoglycosides | Amikacin* | 500 | 375 | 1 | 1 | 3 | 1.83 | 1,404.34 |  |
|  | Gentamicin* | 80 | 382 | 5 | 1 | 2 | 0.54 |  |  |
|  | Colistin | 2 MU | 4.4 MU | 3 | 2 | 9.3 | 24.98 |  |  |
| Carbapenems + colistin + tigecycline | Meropenem | 500 | 1,278 | 3 | 3 | 4 | 6.48 | 4,175.12 |  |
|  | Tigecycline | 50 | 78 | 2 | 2 | 14 | 32.92 |  |  |
|  | Colistin | 2 MU | 4.4 MU | 3 | 2 | 14 | 24.98 |  |  |
| Carbapenems + colistin + Ceftazidime-Avibactam | Meropenem | 500 | 1,278 | 3 | 3 | 3 | 6.48 | 4,375.80 |  |
|  | Ceftazidime | 2,000 | 2,000 | 1 | 3 | 14 | 100.02 |  |  |
|  | Avibactam | 500 | 500 | 1 |  | 14 |  |  |  |
| Carbapenems + colistin + aminoglycosides + tigecycline | Meropenem | 500 | 1,278 | 3 | 3 | 2.5 | 6.48 | 1,139.36 |  |
|  | Tigecycline | 50 | 78 | 2 | 2 | 3.5 | 32.92 |  |  |
|  | Colistin | 2 MU | 4.4 MU | 3 | 2 | 3.5 | 24.98 |  |  |
|  | Gentamicin | 80 | 382 | 5 | 1 | 3 | 0.54 |  |  |

*67% and 33% for amikacin and gentamicin. ^ net cost with lawful discount.

BAT: best available therapy; MU: million units.

**Supplementary Table 4: Estimated hospitalisation costs associated with CRE-KPC infections**

| **Infection** | **DRG** | **Description** | **Duration**  **(days)** | **Tariffs**  **(€)** | **Weight**  **(%)** |
| --- | --- | --- | --- | --- | --- |
| **Basic scenario** | | | | | |
| **Weighted total** |  |  | **12.2** | **4,533.27** | **100%** |
| Hospital acquired pneumonia (HAP)/ Ventilation associated pneumonia (VAP) | 79 | Respiratory infections and inflammations, age > 17 years with CC | 14.9 | 5,744.24 | 8.5% |
| Complicated urinary tract infections (cUTI) | 320 | Kidney and urinary tract infections, age > 17 years with CC | 9.8 | 2,700.58 | 10.6% |
| Complicated intra-abdominal infections (cIAI) | 572 | Major gastrointestinal diseases and peritoneal infections | 10.6 | 3,483.86 | 34.0% |
| Bloodstream infections (BSI) | 576 | Septicaemia without mechanical ventilation ≥ 96 hours, age > 17 years old | 13.3 | 5,492.83 | 46.8% |
| **Alternative scenario (sensitivity analysis)** | | | | | |
| **Weighted total** |  |  | **14.9** | **6,466.51** | **100%** |
| HAP/ Ventilator associated pneumonia (VAP) | 79 | Respiratory infections and inflammations, age > 17 years with CC | 14.9 | 5,744.24 | 8.5% |
| Complicated urinary tract infections (cUTI) | 320 | Kidney and urinary tract infections, age > 17 years with CC | 9.8 | 2,700.58 | 10.6% |
| Complicated intra-abdominal infections (cIAI) | 579 | Post-operative or post-traumatic infections with surgery | 18.6 | 9,163.73 | 34.0% |
| Bloodstream infections (BSI) | 576 | Septicaemia without mechanical ventilation ≥ 96 hours, age > 17 years old | 13.3 | 5,492.83 | 46.8% |

**Supplementary Table 5: Direct costs associated with long-term care**

|  | **Year 1** | **Year 2 and subsequent years** | **Tariffs* (€)** |
| --- | --- | --- | --- |
| Hospitalisation (days) | 12.20 | - | - |
| LTC (days) |  |  |  |
| - up to 30 days | 30.00 | - | 202.00 |
| - beyond the first 30 days | 323.05 | 365.25 | 121.20 |
| **Total (€)** | **€ 45,213.7** | **€ 44,268.3** | **-** |

*"Rehabilitation and long-term hospital stay rates, by type of hospitalisation" published by the Ministry of Health in 2012 [10]

LTC: long-term care

**Supplementary Table 6: Summary of inputs and data sources used to estimate direct costs**

| **Cost item** | **Cost (€)** | **Comments and sources** |
| --- | --- | --- |
| **Acquisition of drugs** | | |
| Best available therapy | 1,485.23 | Composition from TANGO II (validated by an Italian medical expert) and AIFA prices [9] |
| Vaborem | 2,301.49 | Posology and average duration since TANGO II [1] |
| **Pathology management during hospitalisation** | | |
| Hospitalisation | 3,048.04 | Weighted average of DRGs identified for each infection (€ 4,533.27) minus BAT drug acquisition costs (€ 1,485.23)  The proportion of each infection was estimated based on TANGO II data (mCRE-MITT population) [1] |
| **Management of complications associated with the pathology** | | |
| Therapeutic failure | 4,533.27 | Model assumes that patients receive a second course of antibiotic therapy with BAT (€ 1,485.23) and that a second hospitalisation takes place at the same cost as the first (€ 3,048.04) |
| Chronic RRT (annual cost) | 38,819.40 | Roggeri et al. 2016 [11] |
| Long-term hospitalisation–year 1 | 45,213.7 | Rehabilitation and long-term hospital stay rates, by type of hospitalisation" published by the Ministry of Health in 2012 [10] |
| Long-term hospitalisation–after year 1 | 44,268.3 | Rehabilitation and long-term hospital stay rates, by type of hospitalisation" published by the Ministry of Health in 2012 [10] |

AIFA: The Italian Medical Agency; BAT: best available therapy; DRG: diagnosis-related group; mCRE-MITT: microbiologic-CRE-modified intent-to-treat; RRT: renal replacement therapy.

**Supplementary Figure 1: Tornado graph of deterministic sensitivity analyses**


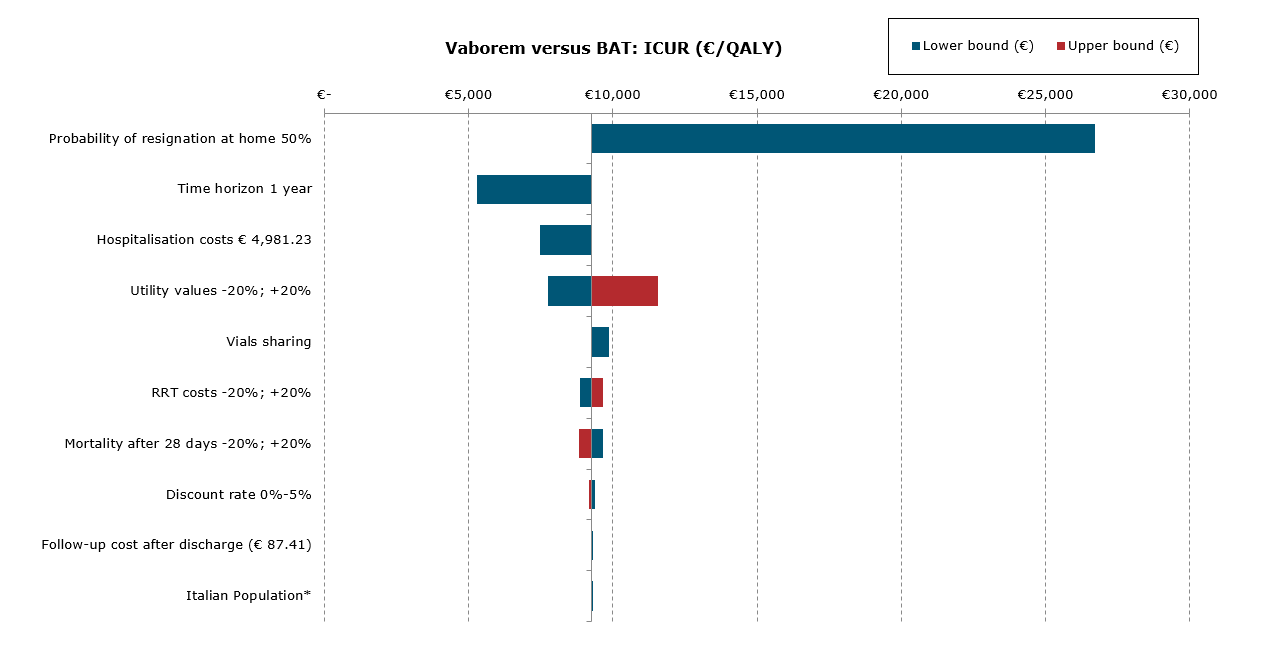


*Patients with an average age of 68.0 years and predominantly male (63.1%) [12].

Note: The scenario where time horizon was set to 28 days could not be represented because its result could not be expressed in terms of ICUR being Vaborem dominant in this scenario.

BAT: best available therapy; ICUR: incremental cost-utility ratio QALY: quality adjusted life year; RRT: renal replacement therapy.

**References**

1. Wunderink RG, Giamarellos-Bourboulis EJ, Rahav G, Mathers AJ, Bassetti M, Vazquez J, et al. Effect and Safety of Meropenem-Vaborbactam versus Best-Available Therapy in Patients with Carbapenem-Resistant Enterobacteriaceae Infections: The TANGO II Randomized Clinical Trial. Infect Dis Ther. 2018;7(4):439-55.

2. Tsevat J, Cook EF, Green ML, Matchar DB, Dawson NV, Broste SK, et al. Health values of the seriously ill. SUPPORT investigators. Ann Intern Med. 1995;122(7):514-20.

3. Ministry Of Health, Attività SDO 2018 2019 [Available from: <http://www.salute.gov.it/imgs/C_17_pubblicazioni_2898_allegato.pdf>.

4. Faulhaber-Walter R, Scholz S, Haller H, Kielstein JT, Hafer C. Health status, renal function, and quality of life after multiorgan failure and acute kidney injury requiring renal replacement therapy. Int J Nephrol Renovasc Dis. 2016;9:119-28.

5. Prescott GJ, Metcalfe W, Baharani J, Khan IH, Simpson K, Smith WC, et al. A prospective national study of acute renal failure treated with RRT: incidence, aetiology and outcomes. Nephrol Dial Transplant. 2007;22(9):2513-9.

6. Liem YS, Bosch JL, Hunink MG. Preference-based quality of life of patients on renal replacement therapy: a systematic review and meta-analysis. Value Health. 2008;11(4):733-41.

7. Bartsch SM, McKinnell JA, Mueller LE, Miller LG, Gohil SK, Huang SS, et al. Potential economic burden of carbapenem-resistant Enterobacteriaceae (CRE) in the United States. Clin Microbiol Infect. 2017;23(1):48 e9- e16.

8. MacNeil Vroomen JL, Boorsma M, Bosmans JE, Frijters DH, Nijpels G, van Hout HP. Is it time for a change? A cost-effectiveness analysis comparing a multidisciplinary integrated care model for residential homes to usual care. PLoS One. 2012;7(5):e37444.

9. Agenzia italiana del Farmaco. Elenco dei Medicinali di fascia A e H. 2019 [Available from: <https://www.aifa.gov.it/en/liste-farmaci-a-h>.

10. Ministry of Health, Italy. Remuneration of hospital care services for acute care, post-acute hospital rehabilitation and long-term care and specialist outpatient care. (13A00528) (OJ General Series n.23 of 28-01-2013 - Ordinary Supplement n.8) 2013 [Available from: <https://www.gazzettaufficiale.it/eli/id/2013/01/28/13A00528/sg>.

11. Roggeri A, Roggeri DP, Zocchetti C, Bersani M, Conte F. Healthcare costs of the progression of chronic kidney disease and different dialysis techniques estimated through administrative database analysis. Journal of nephrology. 2017;30(2):263-9.

12. Tumbarello M, Trecarichi EM, De Rosa FG, Giannella M, Giacobbe DR, Bassetti M, et al. Infections caused by KPC-producing Klebsiella pneumoniae: differences in therapy and mortality in a multicentre study. J Antimicrob Chemother. 2015;70(7):2133-43.
